# Supplementary material for: Plasma dye coating as straightforward and widely applicable procedure for dye immobilization on polymeric materials
Source: Nat Commun. 2018 Mar 16;9:1123. doi: 10.1038/s41467-018-03583-4 (PMC5856759; doi:10.1038/s41467-018-03583-4)
Supplement: Supplementary file 1 — Supplementary Information(PDF 1194 kb) [file 41467_2018_3583_MOESM1_ESM.pdf]

Plasma Dye Coating as Straightforward and Widely Applicable Procedure for Dye Immobilization on Polymeric Materials

Lieselot De Smet,[a#] Gertjan Vancoillie,[a#] Peter Minshall,[a] Kathleen Lava,[a] Iline Steyaert,[b] Ella

Schoolaert,[b] Elke Van De Walle,[c] Peter Dubruel,[c] Karen De Clerck,[b] Richard Hoogenboom[a]*

[a] Supramolecular Chemistry group, Department of Organic and Macromolecular Chemistry, Ghent University, Krijgslaan 281 S4, 9000 Ghent, Belgium, richard.hoogenboom@ugent.be

[b] Centre for Textile Science and Engineering, Department of Materials, Textiles and Chemical Engineering, Faculty of Engineering and Architecture, Ghent University, Technologiepark 907, 9052 Ghent, Belgium.

[c] Polymer Chemistry & Biomaterials Research Group, Department of Organic and Macromolecular Chemistry, Ghent University, Krijgslaan 281 S4Bis, 9000 Ghent, Belgium.

Table of contents

Supplementary Note 1: Chemicals 2
Supplementary Note 2: Methods 2
Supplementary Note 3: Dye modifications 4
- Modification of azobenzene dyes through esterification 4
- Modification of Disperse Red 1 through etherification 5
- Rose Bengal – styrene 7
- 4-N,N'-dimethylamino-1,8-acrylamidoethylnaphthilimide 8
- Sulphonphthaleine modification 10

Supplementary Note 4: PDC parameter optimization 13
- DR1 intensity versus DR1 concentration calibration curve 13
- Optimization of dipping time and dye concentration in dipping solution 13

Supplementary Note 5: Reflective UV-VIS spectroscopy 14
Supplementary Note 6: Brief optimization of plasma time for LD-PE/RB-Sty 15
Supplementary Note 7: Brief optimization of plasma time for UHMW-PE/DR1-A 16
Supplementary Note 8: Surface composition of PDC treated samples 17
Supplementary Note 9: Dye-immobilization and leaching test 20

Supplementary Note 1: Chemicals

The used dyes or starting compounds were used as received with Disperse Red 1 (95%), Disperse Red 13 (95%), Disperse Blue 106 (95%), Solvent Yellow 3 (97%), Bromothymol Blue (95%), Bromocresol Purple (90%) and Methyl Red (crystalline) bought from Sigma-Aldrich and both Rose Bengal (acid red 94, 95%) and 4-bromo-1,8-naphthalic anhydride (95%) bought from TCI Europe. These compounds were modified with (meth)acryloylchloride (97%, 200 ppm MEHQ as stabilizer), vinylbenzylchloride (90%), allylbromide (≥ 98.5%), propargylbromide (≈ 80% in toluene, stabilized with MgO) and 3-aminopropyltriethoxysilane (98%) all bought from Sigma-Aldrich and 4-vinylphenylboronic acid from TCI Europe. $CuSO_4.H_2O$ (≥98%), NaH (60 wt% in mineral oil), dimethylamine (40 wt% in aq.solution), ethylene diamine (≥99.5%), $Pd(PPh_3)_4$ (99%) and N,N'-dicyclohexylcarbodiimide (DCC) were bought from Sigma-Aldrich while tributylvinyltin (97%) was bought from ACROS organics and N-(3-dimethylaminopropyl)-N'-ethylcarbodiimide hydrochloride (EDC.HCl) and $HOBt.H_2O$ from Iris Biotech GmbH. All of these chemicals were used as received, only DCC was purified through dissolving in DCM and drying with $MgSO_4$ (anhydrous, Fischer Chemicals). The electrospinning experiments used polyamide-6 (Mw 51 kDa, Đ 1.82) as base materials and formic acid (> 98%)/acetic acid (99.80%) as solvent all supplied from Sigma-Aldrich. All solvents including dichloromethane, dimethylformamide, n-hexane, ethylacetate, triethylamine and methanol were HPLC-grade from Sigma-aldrich. 1,4-dioxane (HPLC-grade) was bought from ACROS organics and all deuterated solvent from Cambridge Isotopes Laboratories. All anhydrous solvents were obtained by passing over aluminium oxide by means of a J.C. Meyer solvent purification system.

Supplementary Note 2: Methods

Liquid chromatography – mass spectrometry (LCMS). These spectra were measured on an Agilent 1100 HPLC with quaternary pump and UV-DAD detection, coupled to an Agilent G1956B MSD with ESI (multimode) ionization source. 15 µL samples were injected onto a Phenomex-kinetic C18 (5µm 150X4.6mm) column with a flow rate of 1.5 mL/min at 35 °C. most commonly a 6 minute elution

gradient of 0 → 100% acetonitrile in aqueous 5mM ammonium acetate solution was applied. High resolution MS was performed on an Agilent 1100 HPLC with quaternary pump and UV-DAD detection, coupled to an Agilent 6220A TOF-MSD with ESI/APCI (multimode) ionization source.

UV/VIS spectroscopy and reflective UV/VIS spectroscopy. UV/Vis spectra were recorded on a Varian Cary 300 Bio UV-VIS spectrophotometer equipped with a Cary temperature and stir control. Reflective UV/VIS measurements were performed using a Perkin-Elmer Lambda 900 spectrophotometer, which is a double-beam UV-Vis spectrophotometer. For the reflection measurements on fabrics an integrated sphere (Spectralon Labsphere 150 mm) was used. The spectra were recorded from 380 nm to 780 nm with a data interval of 4 nm. Reflection is converted into Kubelka-Munk (K-M) since these values provide a correlation with dye concentration.

Nuclear magnetic resonance (NMR). ^{1}H-NMR and ^{13}C-NMR spectra were recorded on a Bruker Avance 300 MHz or 500 MHz spectrometer at room temperature or an Inova 500 MHz spectrometer. The chemical shifts are given relative to TMS.

X-ray photoelectron spectroscopy (XPS). ESCA S-probe VG monochromatic spectrometer with an Al Kα X-ray source (1486 eV), recorded with a spot size of 250 µm by 1000 µm and analyzed using Casa XPS software package.

Other instruments. Centrifugation was performed on an ALC multispeed refrigerated centrifuge PK 121R from Thermo Scientific using 50 mL centrifuging tubes with screw caps from VWR or 15 mL high clarity polypropylene conical tubes from Falcon. Deionized water was prepared with a resistivity less than 18.2 MΩ x cm using an Arium 611 from Sartorius with the Sartopore 2 150 (0.45 + 0.2 µm pore size) cartridge filter. pH-values were measured using a Consort C561 equipped with a universal pH electrode with build in temperature probe. Fiber morphology of the electrospun structures was examined using a scanning electron microscope (FEI Quanta 200 F) at an accelerating voltage of 20 kV. Sample preparation was done using a gold sputter coater (Balzers Union SKD 030). The nanofiber diameters were measured using UTHSCSA ImageTool version 3.0, developed by the University of

Texas Health Science Center. The average fiber diameters and their standard deviations are based on 50 measurements per sample.

Supplementary Note 3: Dye modifications

Modification of azobenzene dyes through esterification

The synthesis of Disperse Red 1 – acrylate will be discussed as example of the esterification procedure.

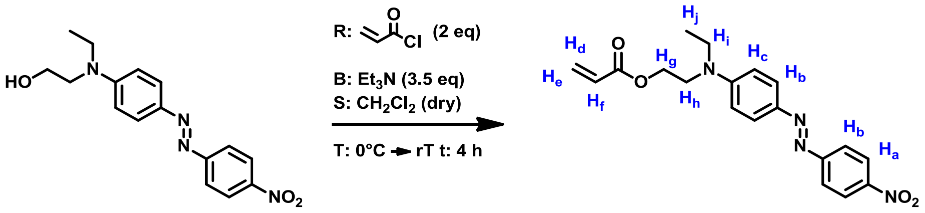

Supplementary Figure 1. Reaction conditions for the synthesis of Disperse Red 1 – acrylate with respective ^{1}H-NMR annotations.

5.09 g of Disperse Red 1 (0.016 mol, 1 eq) was dissolved in anhydrous CH_2Cl_2 (250 mL) and Et_3N (7.7 mL, 0.055 mol, 3.4 eq) and cooled to 0 °C under inert atmosphere. 2.56 mL acryloyl chloride (0.032 mol, 2 eq) was added dropwise to the mixture under vigorous stirring after which the mixture was allowed to warm up to rT. The reaction was monitored using TLC (Silica, CH_2Cl_2/EtOAc 5/1, $Rf_{Reagent}$: 0.43, $Rf_{product}$: 0.89) and was stopped after 4 hours. The product was purified using column chromatography with the same conditions. After drying of the organic phase with $MgSO_4$ the solvent was evaporated, yielding DR1-A as a red powder. **Yield:** 5.688 g (95 %). **^{1}H-NMR spectroscopy (300MHz, CD_2Cl_2)** δ(ppm) 8.24 (2H, d, ^3J = 9.33 Hz, H_a) 7.84 (4H, dd, ^3J = 8.23 Hz, H_b) 6.76 (2H, d, ^3J = 9.88 Hz, H_c) 6.31 (1H, dd, ^3J = 17.56 Hz, ^2J = 2.19 Hz, H_d) 6.05 (1H, dd, ^3J = 18.66 Hz, ^3J = 10.42 Hz, H_e) 5.78 (1H, dd, ^3J = 10.42 Hz, ^2J = 2.19 Hz, H_f) 4.30 (2H, tr, ^3J = 5.49 Hz, H_g) 3.66 (1H, tr, ^3J = 5.49 Hz, H_h) 3.47 (2H, quad, ^3J = 6.58 Hz, H_i) 1.18 (3H, tr, ^3J = 7.68 Hz, H_j).

Disperse Red 1 – methacrylate (DR1-MA). Yield: 1.12 g (92 %). **^{1}H NMR (500 MHz, $CDCl_3$)** δ(ppm) 8.36 (d, J = 9.1 Hz, 2H, H_a), 7.94 (dd, J = 10.9, 9.2 Hz, 4H, H_b), 6.85 (d, J = 9.2 Hz, 2H, H_c), 6.14 (s, 1H, H_d), 5.62 (m, 1H, H_e), 4.41 (t, J = 6.2 Hz, 2H, H_f), 3.77 (t, J = 6.2 Hz, 2H, H_g), 3.58 (q, J = 7.1 Hz, 2H, H_h), 1.97 (s, J = 11.5 Hz, 3H, H_i), 1.29 (t, J = 7.1 Hz, 3H, H_j).

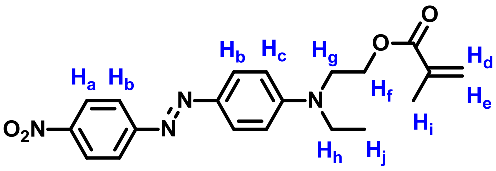

Supplementary Figure 2. Structure of Disperse Red 1 – methacrylate and respective ^{1}H-NMR annotations.

Disperse Red 13 – acrylate (DR13-A). Yield: 0.49 g (85 %). **HR-MS (ESI, TOF):** m/z = 403.1173 ($[M+H]^+$), **^{1}H NMR (300 MHz, $CDCl_3$)** δ(ppm) 8.33 (d, *J* = 2.4 Hz, 1H, H_a), 8.09 (dd, *J* = 8.9, 2.4 Hz, 1H, H_b), 7.89 (d, *J* = 9.3 Hz, 2H, H_c), 7.71 (d, *J* = 8.9 Hz, 1H, H_d), 6.76 (d, *J* = 9.3 Hz, 2H, H_e), 6.36 (dd, *J* = 17.3, 1.4 Hz, 1H, H_f), 6.06 (dd, *J* = 17.3, 10.4 Hz, 1H, H_g), 5.81 (dd, *J* = 10.4, 1.4 Hz, 1H, H_h), 4.32 (t, *J* = 6.3 Hz, 2H, H_i), 3.68 (t, *J* = 6.3 Hz, 2H, H_j), 3.49 (q, *J* = 7.1 Hz, 2H, H_k), 1.20 (t, *J* = 7.0 Hz, 3H, H_l).

Modification of Disperse Red 1 through etherification

The synthesis of Disperse Red 1 – styrene will be discussed as example of the etherification procedure.

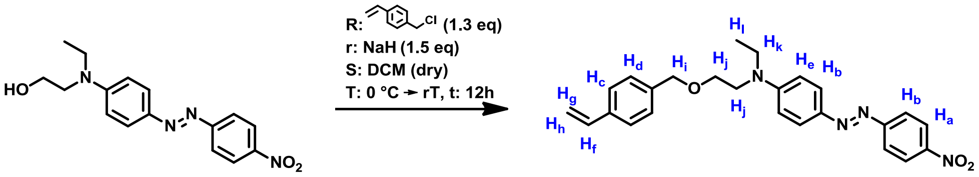

Supplementary Figure 3. Reaction conditions for the synthesis of Disperse Red 1 – styrene (DR1-Sty) with respective ^{1}H-NMR annotations.

54 mg of NaH (60 % suspended, 1.35 mmol, 1.76 eq) was suspended in anhydrous DMF (1 mL) and cooled to 0°C. 0.241 g of DR1 (0.77 mmol, 1 eq) was dissolved in 1.5 mL DMF and added slowly to the mixture while stirring vigorously at 0°C for 15min. subsequently, 0.17 mL of vinylbenzylchloride (1.09 mmol, 1.42 eq) was added dropwise after which the mixture was allowed to heat up to rT and stirred overnight. The reaction was monitored using TLC (Silica, DCM/n-Hex 8/2) which showed nearly complete consumption of DR1 after 12h. Residual NaH was deactivated by slowly diluting the reaction mixture with 5 mL of sat. NH_4Cl at 0°C after which the compounds were extracted with EtOAc. The organic fractions were collected, washed with brine, dried with Na_2SO_4 and dried under vacuum. The resulting dark red shiny solid was further purified using column chromatography (Silica, DCM/n-Hex 8/2) yielding DR1-Sty as a red powder. **Yield:** 0.15 g (44 %), **HR-MS (ESI, TOF):** m/z = 431.2078 ($[M+H]^+$, Δ < 1ppm), 453.1897 ($[M+Na]^+$, < 1ppm). **^{1}H-NMR spectroscopy (500 MHz, $CDCl_3$):**

δ (ppm) 8.47 – 8.26 (m, 2H, H_a), 8.01 – 7.84 (m, 4H, H_b), 7.41 (d, J = 8.1 Hz, 2H, H_c), 6.86 – 6.64 (m, 3H, $H_{e\text{-}f}$), 5.77 (d, J = 17.6 Hz, 1H, H_g), 5.27 (d, J = 10.9 Hz, 1H, H_h), 4.56 (s, 2H, H_i), 3.78 – 3.62 (m, 4H, H_j), 3.57 (q, J = 7.2 Hz, 2H, H_k), 1.26 (t, J = 7.1 Hz, 3H, H_l). (H_d overlaps with the $CDCl_3$ solvent signal)

Disperse Red 1 – allyl (DR1-allyl). Yield: 0.49 g (87.5 %), **LC-MS (ESI):** m/z = 355.20 ($[M+H]^+$). **^{1}H-NMR spectroscopy (300 MHz, $CDCl_3$):** δ (ppm) 8.25 (d, *J* = 9.0 Hz, 2H, H_a), 7.86 (dd, *J* = 9.2, 2.4 Hz, 4H, H_b), 6.72 (d, *J* = 9.2 Hz, 2H, H_c), 5.83 (ddd, *J* = 22.5, 10.6, 5.5 Hz, 1H, H_d), 5.21 (2dd, *J* = 17.2, 1.4 Hz, 2H, H_e), 3.94 (d, *J* = 5.5 Hz, 2H, H_f), 3.59 (s, 4H, H_g), 3.50 (q, *J* = 7.1 Hz, 2H, H_h), 1.19 (t, *J* = 7.1 Hz, 3H, H_i).

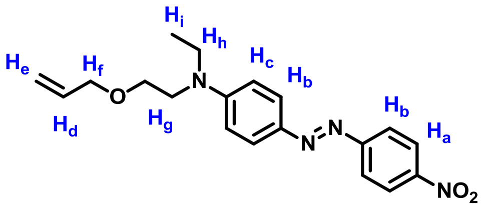

Supplementary Figure 4. Structure of Disperse Red 1 – allyl and respective ^{1}H-NMR annotations.

Disperse Red 1 – propargyl (DR1-propargyl). Yield: 0.34 g (63 %), **HR-MS (ESI, TOF):** m/z = 353.1624 ($[M+H]^+$, Δ 4.5ppm). **^{1}H-NMR spectroscopy (500 MHz, Acetone-6d):** δ (ppm) 8.39 – 8.13 (m, 2H, H_a), 7.96 – 7.82 (m, 2H, H_b), 7.82 – 7.71 (m, 2H, H_c), 6.87 – 6.72 (m, 2H, H_d), 4.0**9** (d, *J* = 2.4 Hz, 2H, H_e), 3.72 – 3.55 (m, 4H, H_f), 3.50 (q, *J* = 7.1 Hz, 2H, H_g), 2.84 (t, *J* = 2.4 Hz, 1H, H_h), 1.18 – 1.07 (m, 3H, H_i). **^{13}C-NMR spectroscopy (APT, 500 MHz, Acetone-6d):** δ(ppm) 156.87 (C_k), 151.97 (C_m), 147.48 (C_j), 143.43 (C_l), 126.14 (C_c), 124.70 (C_a), 122.48 (C_b), 111.60 (C_d), 75.22 (C_h), 67.40 (C_{f1}), 57.95 (C_e), 49.90 (C_{f2}), 45.61 (C_g), 11.54 (C_i).

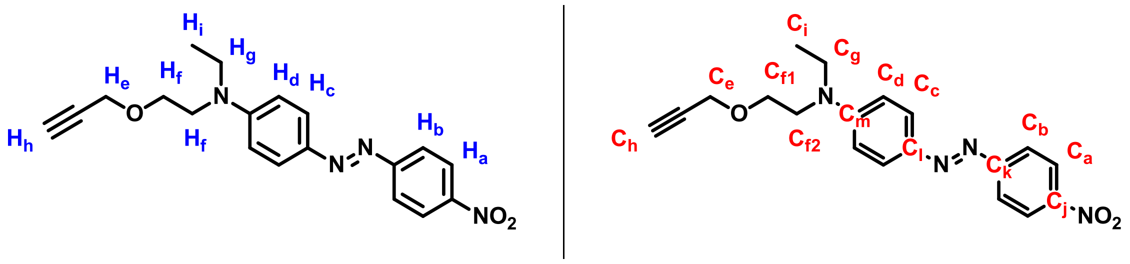

Supplementary Figure 5. Structure of Disperse Red 1 – propargyl and respective ^{1}H-NMR and ^{13}C-NMR annotations.

Rose Bengal – styrene

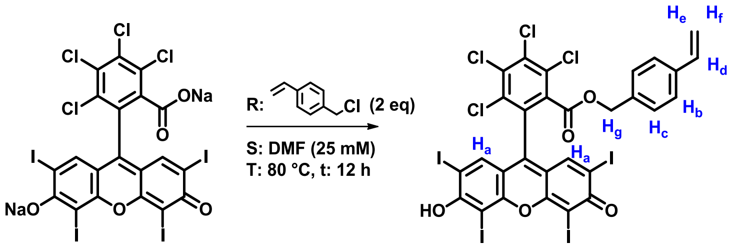

Supplementary Figure 6. Reaction conditions for the synthesis of Rose Bengal – styrene (RB-Sty) with respective ^{1}H-NMR annotations.

Rose Bengal (0.536 g, 0.53 mmol, 1 eq) and a spatula point of phenothiazine was dissolved in 20 mL of DMF (anhydrous) under Ar atmosphere. To this mixture, 0.16 mL of 4-chloromethylstyrene (1.14 mmol, 2.16 eq) was added after which the temperature was raised to 80°C and stirred overnight. The reaction was followed using TLC (Silica, DCM/MeOH 95/5, $R_{f,RB}$ = 0, $R_{f,RB\text{-}Sty}$ = 0.1) and was purified by evaporating the DMF under reduced pressure and stirring the resulting solid in diethyl ether for 6h. After filtration, the solid was purified using column chromatography (Silica, DCM/MeOH 95/5) yielding RB-Sty as a shiny, purple solid after evaporation. **Yield:** 0.447 g (78 %). **HR-MS (ESI, TOF):** m/z 1088.5673 ($[M+H]^+$, Δ 1.6 ppm). **^{1}H-NMR spectroscopy (300 MHz, DMSO-6d)** δ(ppm) 7.54 (s, *J* = 5.5 Hz, 2H, H_a), 7.23 (d, *J* = 8.2 Hz, 2H, H_b), 6.80 (d, *J* = 8.2 Hz, 2H, H_c), 6.68 (dd, *J* = 17.7, 10.9 Hz, 1H, H_d), 5.82 (dd, *J* = 17.7, 0.9 Hz, 1H, H_e), 5.27 (dd, *J* = 10.9, 0.9 Hz, 1H, H_f), 5.02 (s, 2H, H_g).

4-N,N'-dimethylamino-1,8-acrylamidoethylnaphthilimide

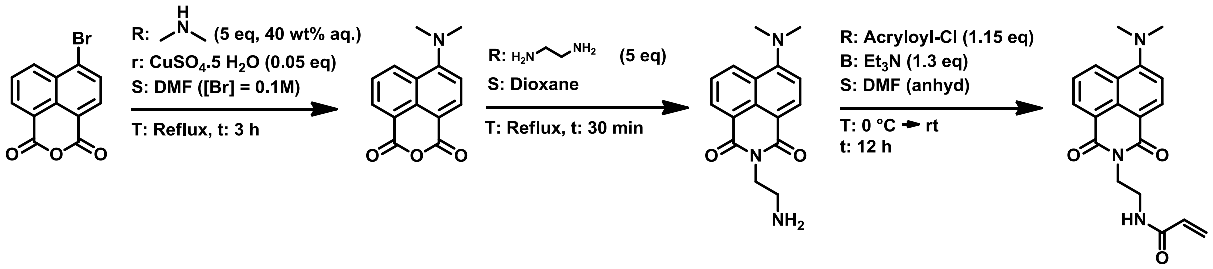

Supplementary Figure 7. Full synthetic scheme for the synthesis of 4-*N,N'*-dimethylamino-1,8-acrylamidoethylnaphthilimide (DMAAENI).

Step 1. Synthesis of 4-*N,N'*-dimethylamino- 1,8-naphthalic anhydride (DMANA)

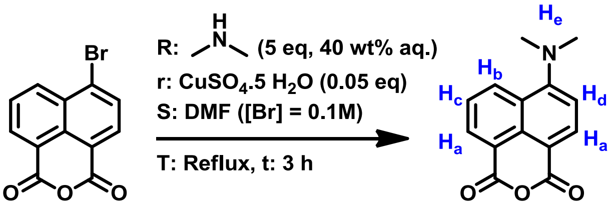

Supplementary Figure 8. Reaction mechanism for the synthesis of 4-N,N'-dimethylamino-1,8-naphthilic anhydride with respective ^{1}H-NMR annotations.

5.02 g of 4-bromo-1,8-naphthalic anhydride (0.018 mol, 1 eq) was dissolved in a mixture of 12 mL dimethylamine (40 wt% aqueous solution, 0.095 mol, 5.2 eq) and 30 mL of DMF. 0.22 g of $CuSO_4$ pentahydrate (0.89 mmol, 0.05 eq) was added after which the mixture was refluxed for 4 hours. Upon cooling of the reaction mixture to rT the compound precipitated. After filtration, the yellow powder was dried in a vacuum oven overnight. **Yield:** 3.22 g (73.56 %). **^{1}H-NMR spectroscopy ($CDCl_3$, 300 MHz)** δ(ppm) 8.46 (2dd, *J* = 9.7, 7.9 Hz, 2H, H_a), 8.38 (d, *J* = 8.3 Hz, 1H, H_b), 7.61 (dd, *J* = 8.5, 7.3 Hz, 1H, H_c), 7.05 (d, *J* = 8.3 Hz, 1H, H_d), 3.11 (s, *J* = 2.3 Hz, 6H, H_e).

Step 2. Synthesis of 4-*N,N'*-dimethylamino-1,8-aminoethylnaphthilimide (DMAENI)

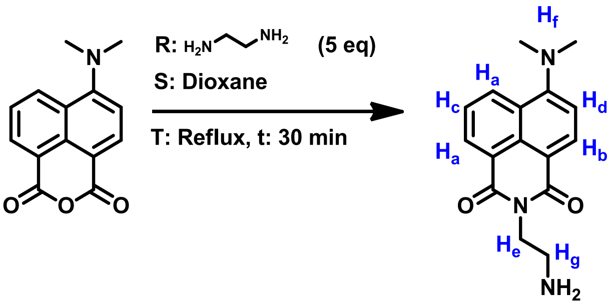

Supplementary Figure 9. Reaction mechanism for the synthesis of 4-N,N'-dimethylamino-1,8-aminoethylnaphthilamide with respective ^{1}H-NMR annotations.

0.96 g of DMANA (3.99 mmol, 1 eq) was dispersed in 10 mL of 1,4-dioxane and added dropwise to a refluxing mixture of 1.4 mL of ethylene diamine (20.9 mmol, 5.24 eq) and 30 mL of 1,4-dioxane. Upon addition of the DMANA solution, the mixture became clear and after full addition it was stirred and refluxed for another hour. After evaporation of the solvent, the residual yellowish solid was purified using a gradient silica column. To remove any residual DMANA or double reacted product, the column was washed with DCM/MeOH 99/1 to 97/3, after which the compound was retrieved by washing with 1% of EtN_3 in DCM/MeOH 97/3. After removal of the solvent, the product was obtained as a yellow powder. **Yield:** 0.62 g (55%). **^{1}H-NMR spectroscopy (300 MHz, MeOD)** δ(ppm) 8.45 (m, 2H, H_a), 8.33 (d, J = 8.3 Hz, 1H, H_b), 7.65 (dd, J = 8.5, 7.4 Hz, 1H, H_c), 7.14 (d, J = 8.3 Hz, 1H, H_d), 4.19 (t, J = 6.6 Hz, 2H, H_e), 3.11 (s, 6H, H_f), 2.95 (t, J = 6.6 Hz, 2H, H_g). **^{13}C-NMR spectroscopy (300 MHz, MeOD)** δ(ppm) 166.31 (C_h), 165.78 (C_h), 158.78 (C_i), 133.83 (C_b), 132.94 (C_a), 131.99 (C_a), 131.50 (C_j), 126.26 (C_j), 125.84 (C_c),123.84 (C_j), 115.12 (C_j), 114.20 (C_d), 45.03 (C_f), 43.10 (C_e), 40.96 (C_g).

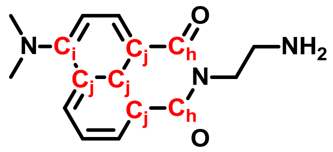

Supplementary Figure 10. Structure of 4-N,N'-dimethylamino-1,8-aminoethylnaphthilamide with ^{13}C-NMR annotations of quaternary carbons.

Step 3. Synthesis of 4-N,N'-dimethylamino-1,8-acrylamidoethylnaphthilimide (DMAAENI)

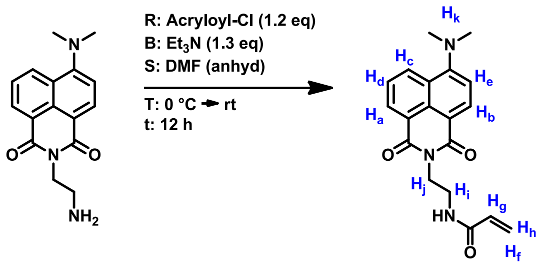

Supplementary Figure 11. Reaction mechanism for the synthesis of 4-N,N'-dimethylamino-1,8-acrylamidoethylnaphthilamide with respective ^{1}H-NMR annotations.

0.50 g of DMAENI (1.75 mmol, 1 eq) was dissolved in a mixture of 0.32 mL of triethylamine (2.29 mmol, 1.31 eq) with 20 mL of dry DMF and cooled to 0 °C. 0.17 mL of acryloylchloride (2.10 mmol, 1.20 eq) was diluted in 5 mL of dry DMF and added slowly to the cooled mixture while stirring. After addition, the mixture was allowed to heat up to room temperature and stirred overnight. The

reaction was followed using TLC (silica, DCM/Acetone 8/2, Rf = 0.41) and after all the amine reacted, the solvent was evaporated. The solid was purified using column chromatography yielding the product as a bright yellow powder. **Yield:** 0.25 g (42.2 %). **HR-MS (ESI-TOF):** m/z 338.119 (Δ 0.4 ppm). **^{1}H-NMR spectroscopy (400 MHz, $CDCl_3$)** δ (ppm) 8.57 (dd, *J* = 7.3, 1.2 Hz, 1H, H_a), 8.52 – 8.40 (d + dd, *J* = 8.2 Hz + 8.5, 1.2 Hz, 2H, H_b + H_c), 7.65 (dd, *J* = 8.5, 7.3 Hz, 1H, H_d), 7.10 (d, *J* = 8.3 Hz, 1H, H_e), 6.17 (dd, *J* = 17.1, 1.6 Hz, 1H, H_f), 6.06 (dd, *J* = 17.1, 10.1 Hz, 1H, H_g), 5.56 (dd, *J* = 10.1, 1.6 Hz, 1H, H_h), 4.52 – 4.36 (m, 2H, H_i), 3.83 – 3.63 (m, 2H, H_j), 3.12 (s, 6H, H_k). **^{13}C-NMR spectroscopy (400 MHz, $CDCl_3$)** δ(ppm) 166.05 (C_l), 165.51 (C_l), 165.05 (C_l), 157.51 (C_m), 133.27 (C_b), 131.80 (C_c), 131.54 (C_a), 131.29 (C_g), 130.61 (C_n), 125.86 (C_f &C_h), 125.27 (C_n), 124.97 (C_d), 122.78 (C_n), 114.36 (C_n), 113.36 (C_e), 44.90 (C_k), 40.49 (C_j), 39.18 (C_i).

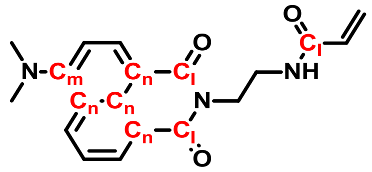

Supplementary Figure 12. Structure of 4-N,N'-dimethylamino-1,8-aminoethylnaphthilamide with ^{13}C-NMR annotations of quaternary carbons.

Sulphonphthaleine modification

Suzuki coupling with Bromothymol Blue

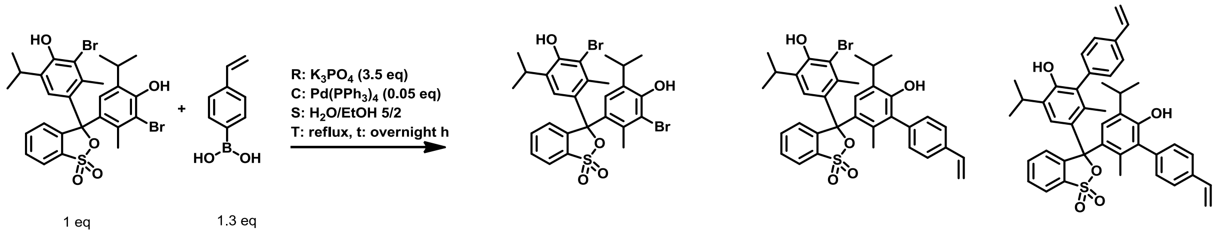

Supplementary Figure 13. Modification of Bromothymol Blue with styrene moieties using Suzuki coupling.

0.501 g of Bromothymol Blue (BTB, 0.813 mmol, 1 eq), 0.153g of 4-vinylphenyl boronic acid (4-VBA, 1.035 mmol, 1.27 eq) and 0.612 g of K_3PO_4 (2.88 mmol, 3.5 eq) were dissolved in 8 mL of a 7/3 mixture of water/EtOH. This dark blue solution was degassed using three freeze-pump-thaw cycles to remove all traces of oxygen after which 46.27 mg of $Pd(PPh_3)_4$ (0.004 mmol, 0.05 eq) was added as a

solid under a slight argon overpressure. The resulting suspension was stirred and heated to reflux (80 °C) overnight. The mixture was purified by filtering off the $Pd(PPh_3)_4$, acidifying with 1M HCl and extraction with EtOAc. The resulting colored solid contained BTB, single reacted BTB-Sty and double reacted BTB-Sty². **LC-MS (kinetex C18, ACN 0 → 100% in 6 min, ESI)** t_1: 5.248 min, m/z 625.00 ($[BTB+H]^+$); t_2 = 5.771 min, m/z 648.10 ($[BTB\text{-}Sty+H]^+$); t_3 = 6.132 min, m/z 670.20 ($[BTB\text{-}Sty^2+H]^+$). BTB-Sty was isolated using reverse phase preparative HPLC. **Prep-HPLC (kinetex C18, H_2O+0.1%TFA/ACN 50 → 100% in 30 min + 5 min 100% ACN, flow rate 17.5mL)** t_1: 15.401 min (BTB); t_2: 23.459 min (BTB-Sty); t_3: 32.922 min (BTB-Sty²). Peaks were identified using LC-MS as described above. **Yield:** 11.1 mg (1.9 %). Using higher temperature (140°C in the µW), shorter reaction time (3h) and large excesses of 4-VBA (3 eq) and K_3PO_4 (16.5 eq), the formation of the double reacted product was preferred, which could subsequently be isolated by normal phase column chromatography on silica using CHCl3/MeOH 5/1 with 1% FA. **Yield:** 64.2mg (11%). **HR-MS (ESI, TOF):** m/z = 669.2698 ($[BTB\text{-}Sty^2 + H]^+$ $\Delta < 2.7$ppm). ^{1}H-NMR spectroscopy showed a complex mixture of various isomers resulting from the pH responsivity and possible keto-enol equilibrium.

Stille coupling with Bromocresol Purple

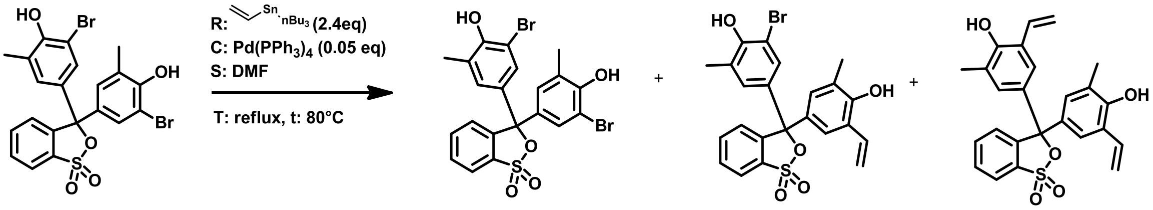

Supplementary Figure 14. Synthetic scheme of the Stille coupling of Bromocresol Purple with tributylvinyltin.

0.2502g of Bromocresol Purple (0.46 mmol, 1 eq) and 0.0295g of $Pd(PPh_3)_4$ (0.02 mmol, 0.05 eq) were dissolved in 10 mL of DMF (0.12 mol) which was bubbled with Ar for 30 min. A spatula point of phenothiazine was added to prevent self-initiation of the formed styrene moiety. 0.31 mL tributyl(vinyl)tin (1.10mmol, 2.4 eq) was added after which the reaction was heated to 80°C and stirred overnight. The reaction was purified by evaporating of the DMF through azeotropic evaporation with toluene after which the crude product was purified using column chromatography (silica, MeOH/DCM 1/5) revealing a deep red solid after evaporation. LC-MS revealed that the solid

contained three main products namely double reacted BCP (BCP-vinyl²), single reacted BCP(BCP-vinyl) and unreacted BCP. **LC-MS (kinetex C18, ACN 0 → 100% in 6 min, ESI)** t_1: 4.575 min, m/z 539.80 ([BCP-H]$^-$); t_2 = 4.725 min, 486.90 m/z ([BCPvinyl-H]$^-$); t_3 = 4.879 min, 433.26 m/z ([BCPvinyl²-H]$^-$). The addition of an excess of tributylvinyl reagent caused the predominant formation of BCPvinyl² in the presence of unreacted BCP allowing its use in the plasma dye coating experiments. Equimolar addition of tributylvinyltin showed almost exclusive formation of BCPvinyl in the presence of BCP, which will be further purified using reverse phase preparative HPLC in future work. ^{1}H-NMR spectroscopy showed a complex mixture of various isomers resulting from the pH responsivity and possible keto-enol equilibrium.

Supplementary Note 4: PDC parameter optimization

DR1 intensity versus DR1 concentration calibration curve

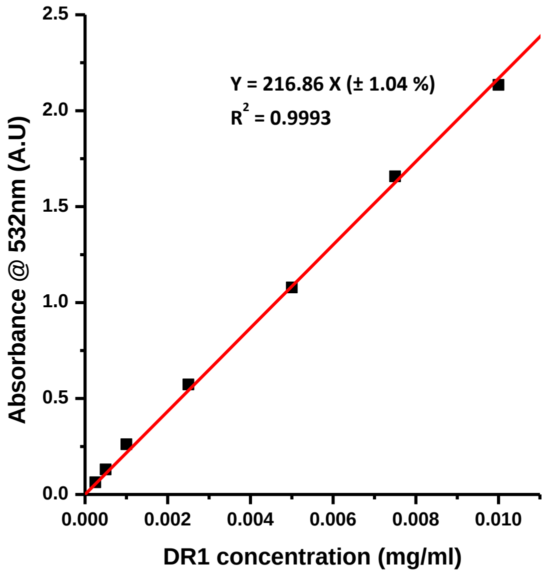

Supplementary Figure 15. Calibration curve of DR1 absorbance at 532nm in 50/50 FA/AA.

Optimization of dipping time and dye concentration in dipping solution

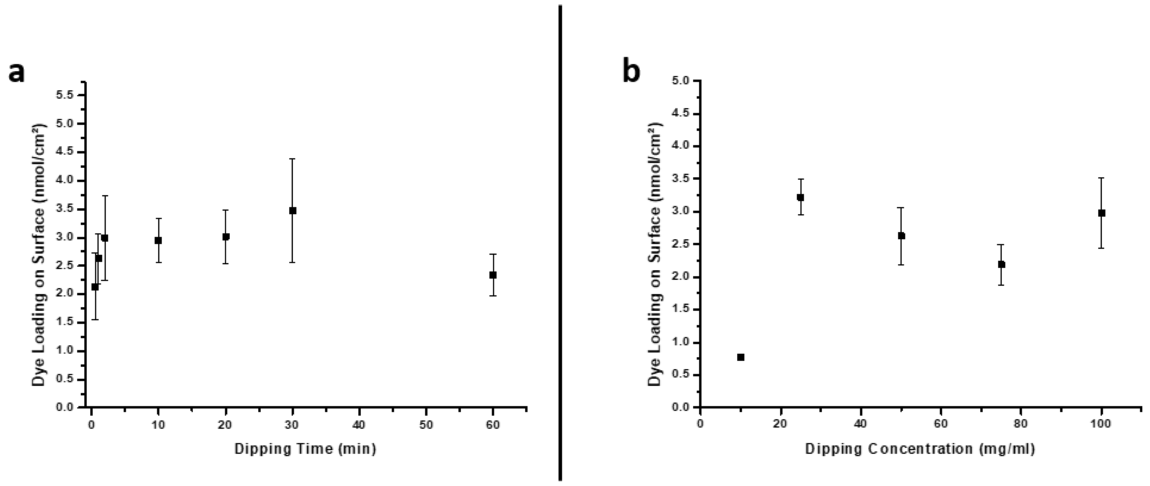

Supplementary Figure 16. Dye loading on PA6 surface after PDC treatment measured by UV-VIS spectroscopy in 1/1 FA/AA in function of dipping time (a) and dipping concentration (b).

Supplementary Note 5: Reflective UV-VIS spectroscopy

A quantitative characterisation of the dye loading of DR1-A on different PDC modified materials, prepared by using the above optimized conditions, was possible via reflective UV-VIS spectroscopy (Supplementary Figure 17). All materials were treated with DR1-A and immobilized under optimized PDC conditions (plasma time: 1 min, dipping concentration: 25 mg/ml, dipping time: 1 min). The highest dye loading was observed for PA6, which was expected as the process was optimized for PA6. As the surface area of fibrous cellulose and PA6 is much higher than for the non-fibrous LD-PE and Teflon, more dye molecules can be covalently linked onto the material, leading to a higher color intensity which is confirmed in the absorbance graphs. The necessity of the plasma step in the PDC procedure is also demonstrated in Supplementary Figure 17. Almost no difference in intensity is noticed between the counter sample without the necessary plasma treatment (PDC without plasma) and the non-treated sample (untreated material).

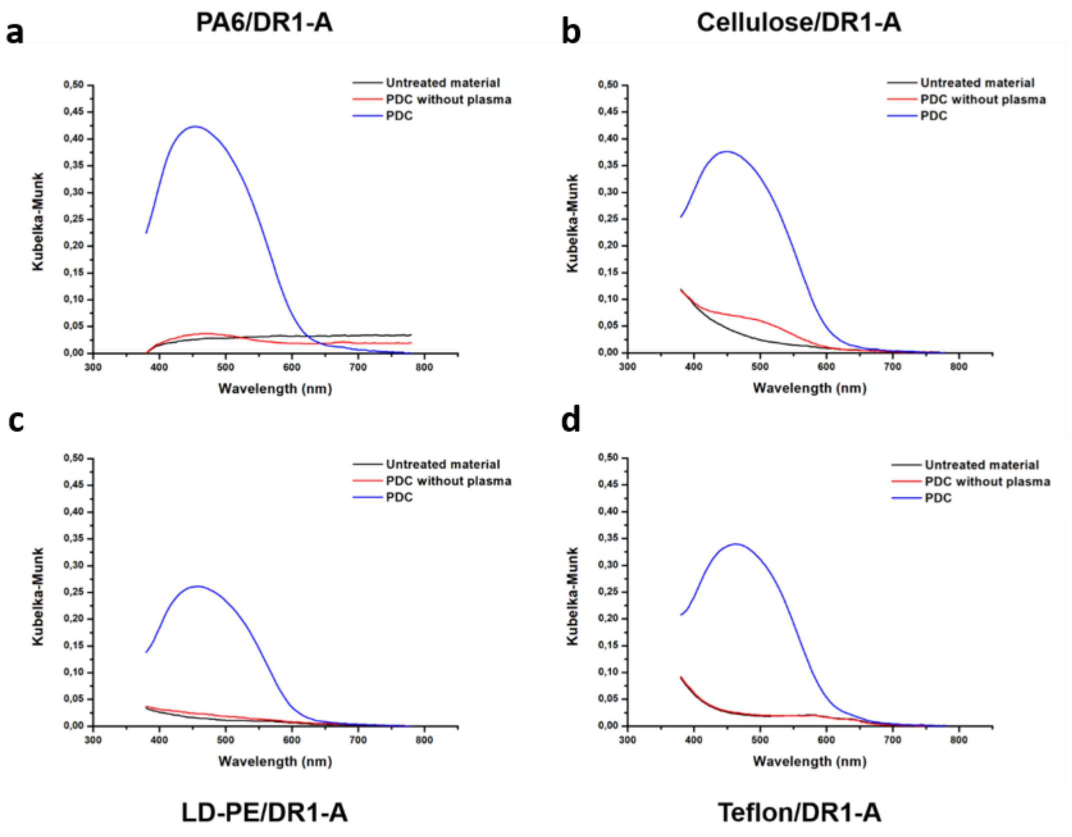

Supplementary Figure 17. The Kubelka-Munk results, normalized at maximum reflection, are given for non-treated (Untreated material), PDC without the plasma treatment step (PDC without plasma) and a PDC treated (PDC) sample of PA6/DR1-A (a), Cellulose/DR1-A (b), LD-PE/DR1-A (c) and Teflon/DR1-A (d).

Supplementary Note 6: Brief optimization of plasma time for LD-PE/RB-Sty

When using the optimized PDC parameters as described in the paper for the immobilization of RB-sty on LD-PE, the coloration is difficult to notice (Supplementary Figure 18). In order to further validate our claim of dye-material specificity in the PDC procedure, a quick optimization of the plasma time for the LD-PE/RB-Sty combination was performed. This confirms that a plasma time of 1 minute on each side indeed results in limited coloration of the material while a slight increase in color was visually noticed with longer plasma times, see Supplementary Figure 18 (Top). This trend was confirmed via reflective UV-VIS (Supplementary Figure 18, Bottom) and a maximum absorbance is reached after a plasma time of 2.5 min for LD-PE/RB-Sty with a dipping concentration of 25 mg/ml and a dipping time of 1 min. This however indicates that although the PDC procedure is widely applicable due to the non-specific nature of the immobilization process, a specific combination of dye and possibly the functional group with the chosen material could require their own set of optimized parameters.

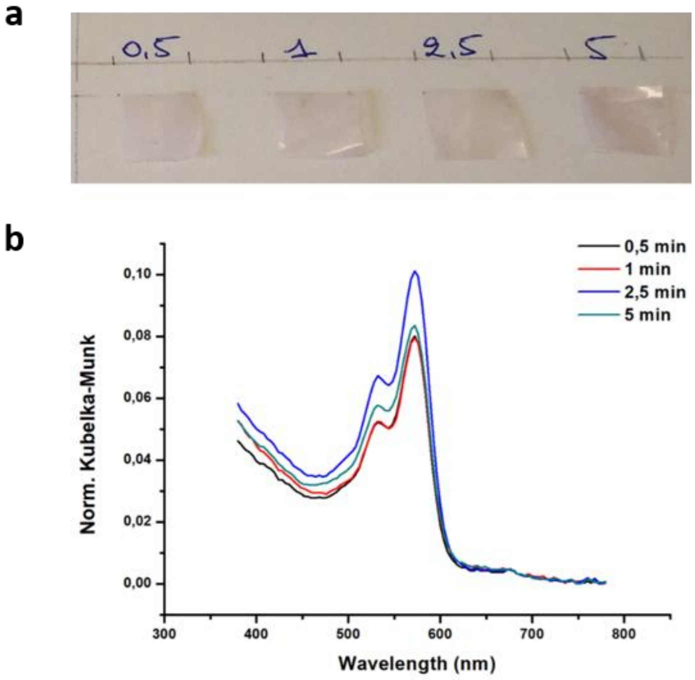

Supplementary Figure 18. (a) Optimization of the plasma time on each side of the material for the RB-LD-PE combination. (b) Kubelka-Munk values, normalized at max reflection, for LD-PE/RB-Sty treated respectively 0.5 min, 1 min, 2.5 min or 5 min plasma time.

Supplementary Note 7: Brief optimization of plasma time for UHMW-PE/DR1-A

The plasma time for UHMW-PE/DR1-A was optimized using reflective UV-VIS (Supplementary Figure 19). A significant increase in color intensity is noticed with plasma times higher than 1 min and a maximum absorbance is reached after a plasma time of 4 min for UHMW-PE/DR1-A with a dipping concentration of 25 mg/ml and a dipping time of 1 min.

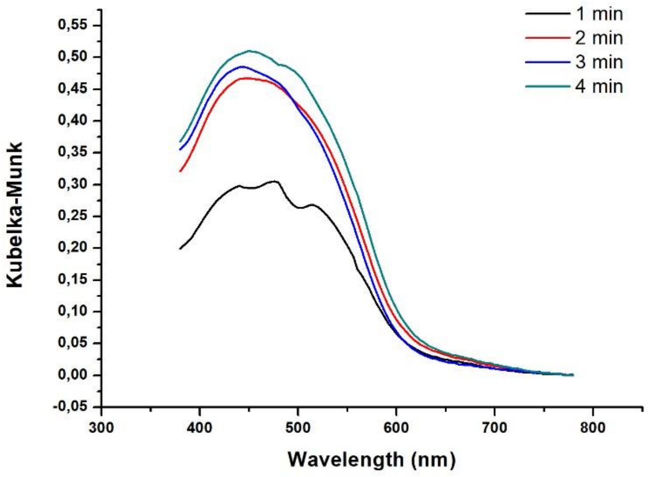

Supplementary Figure 19. Optimization of the plasma time of UHMW-PE fibers with DR1-A. Kubelka-Munk values, normalized at max reflection, for UHMW-PE/DR1-A treated respectively 1 min, 2 min, 3 min or 4 min plasma time.

Supplementary Note 8: Surface composition of PDC treated samples

The effect of the plasma treatment on the material surface composition was investigated using contact angle measurements with a water droplet. Values for untreated surfaces were compared to samples modified with the hydrophobic DR1 and the hydrophilic RB, immobilized using the PDC procedure for PA-6, cellulose, PP, PTFE, LD-PE and HD-PE. All tests were performed in triplicate by placing a drop of MilliQ water on each surface after which the contact angle was determined after 5 sec, averaged over five drops per sample

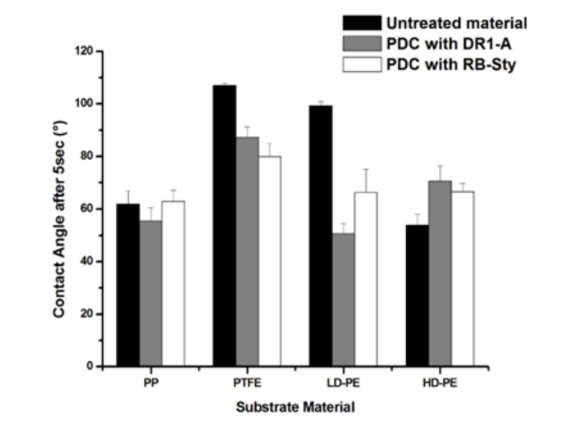

Supplementary Figure 20. Effect of PDC on the surface properties and composition. (Top) Contact angle measurements of a water droplet. The contact angle of the water droplet is measured after 5 sec on the material surface for untreated material (black), PDC treated material with DR1-A (grey) and PDC treated material with RB-Sty (white). A drop of MilliQ water was placed on each surface after which the contact angle was determined after 5 sec, averaged over five drops per sample. The reproducibility of all samples was confirmed by measuring the contact angles in triplicate.

The effect of the plasma treatment on the material surface composition was further investigated by using X-ray photoelectron spectroscopy (XPS). The atomic ratios of the untreated material were compared with PDC treated samples with varying plasma times (PA6 and LD-PE) or optimized PDC conditions (cellulose, Teflon and UHMWPE). All investigations were performed with RB-Sty and include a reference example that underwent the same PDC procedure, but without final plasma treatment (Supplementary Figure 22). Important to note is that all inert materials (LD-PE, Teflon and UHMWPE) were pretreated to ensure adsorption of the dye on the surface. Firstly, no traces of the dye, indicated by the presence of I or Cl atoms, could be observed indicating too the low loading of dye on the surface (nmol/cm²), despite that the color of the materials and the reflective UV-vis spectroscopy confirmed the successful immobilization of the dye after soxhlet extraction. Secondly,

no noticeable difference in O/C ratio is observed between untreated samples and PDC treated samples, meaning that the PDC procedure has little to no influence on the surface composition at reasonable plasma time (< 5min) for PA6, LDPE, cellulose and UHMWPE. Further increase in plasma time for PA6 shows an increase in O/C ratio, most likely resulting from exposure to air immediately after plasma treatment. Finally, the pretreatment step of Teflon removes all traces of fluoride on the surface indicated by the decreased ratio of the PDC without plasma sample. The success of the PDC procedure for this material has been visually confirmed but more investigations towards the possible changes in surface properties for Teflon need to be performed.

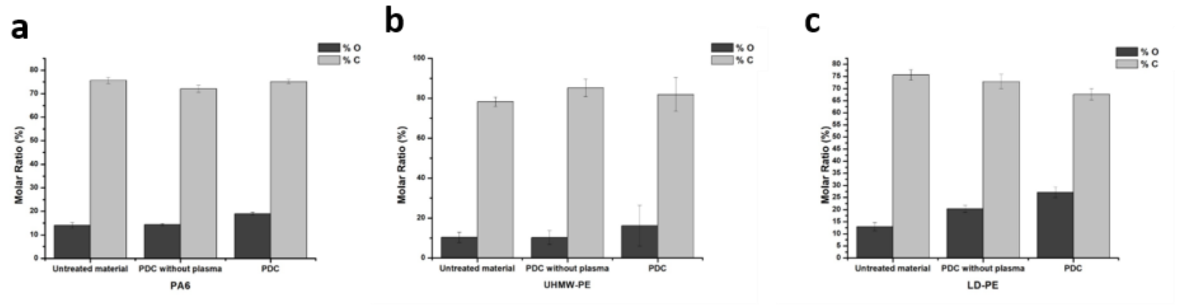

Supplementary Figure 21. X-ray photoelectron spectroscopy measurements. The results for PA6 (a), UHMW-PE (b) and LD-PE (c) are given as the carbon and oxygen ratios for untreated (untreated material), PDC without the plasma treatment step (PDC without plasma) and the PDC treated (PDC) sample. All tests were performed on a 1 cm^2 piece of material in triplicate.

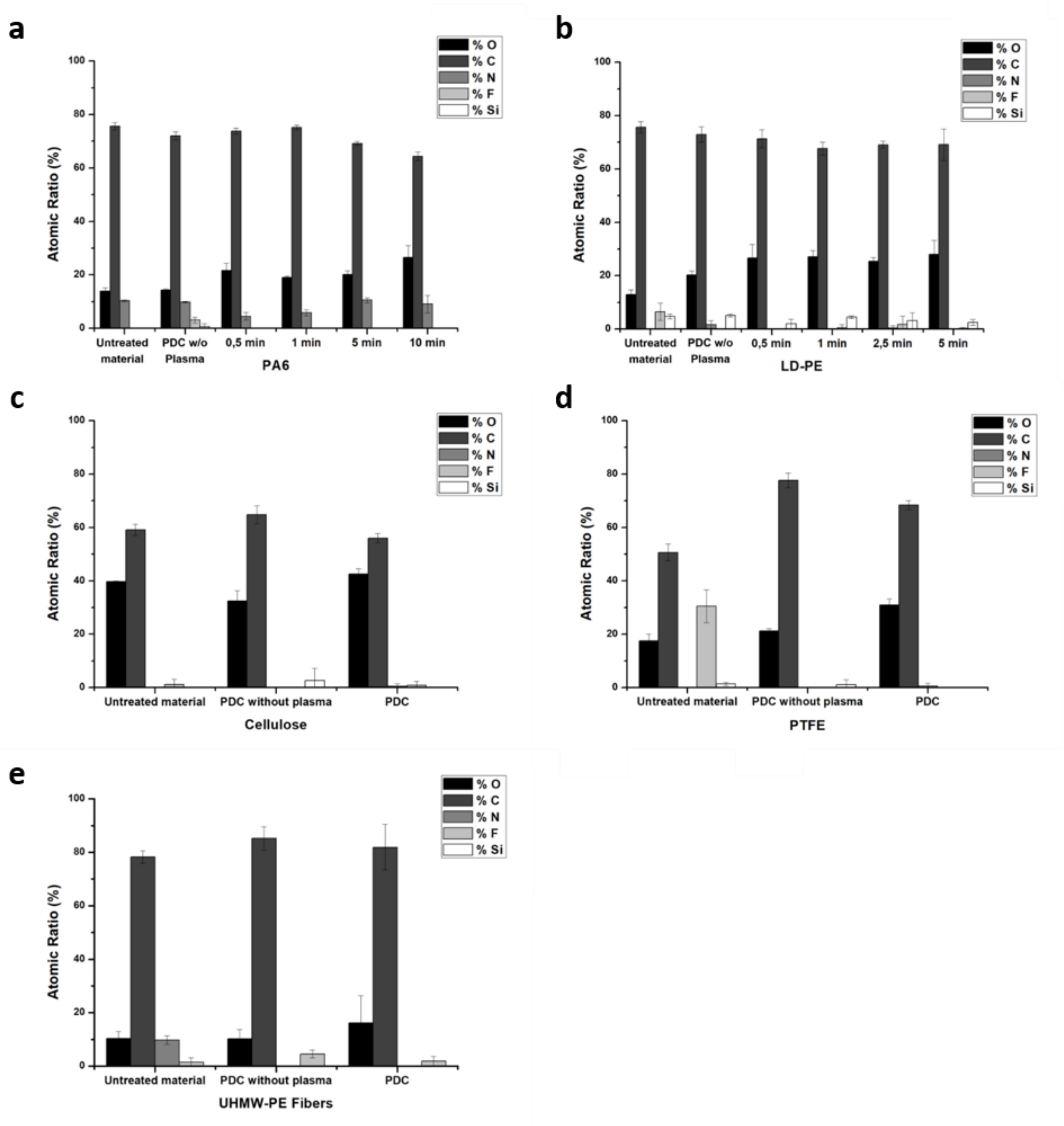

Supplementary Figure 22. Results XPS measurements. Top: The surface atomic ratios of PA6 (a) and LD-PE (b) untreated (Untreated material), PDC without the plasma treatment step (PDC without (w/o) plasma) and the PDC treated sample with increasing plasma time. 1) PA6: 0.5 min, 1 min, 5 min and 10 min respectively. 2) LPDE: 0.5 min, 1 min, 2.5 min and 5 min. bottom: The surface atomic ratios of Cellulose (c), PTFE (d) and UHMWPE fibers (e): untreated (Untreated material), PDC without the plasma treatment step (PDC without plasma) and the PDC treated (PDC) sample. *The nitrogen content is probably a contaminant.

Supplementary Note 9: Dye-immobilization and leaching test

Color measurements were performed using a Perkin-Elmer Lambda 900 spectrophotometer, which is a double-beam UV-Vis spectrophotometer, equipped with an integrated sphere (Spectralon Labsphere 150 mm) allowing for the reflection measurements on fabrics. The spectra were recorded from 380 nm to 780 nm with a data interval of 4 nm and converted into Kubelka-Munk (K-M).

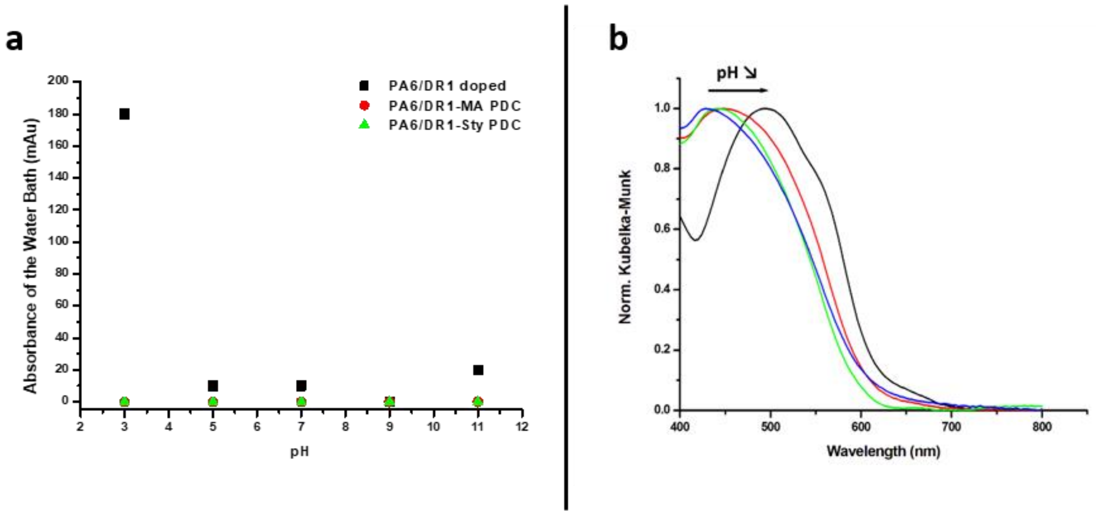

Supplementary Figure 23. (a) Water fastness tests reflecting the dye migration to a water bath by plotting the absorbance at the maximum wavelength in function of the pH for a PA6/DR1 doped sample (black squares), PA6/DR1-MA treated with PDC (red circles) and a PA6/DR1-Sty treated with PDC (green triangles). (b) Normalized Kubelka-Munk values of the DR1-A/PA6 samples, illustrating the color change from bright pink to orange through a hypsochromic shift with increasing pH from 0 (black), 1 (red), 2 (green) and 12 (blue).
