# Supplementary material for: Plasma dye coating as straightforward and widely applicable procedure for dye immobilization on polymeric materials
Source: Nat Commun. 2018 Mar 16;9:1123. doi: 10.1038/s41467-018-03583-4 (PMC5856759; doi:10.1038/s41467-018-03583-4)
Supplement: Supplementary file 3 — Description of Additional Supplementary Files(PDF 50 kb) [file 41467_2018_3583_MOESM3_ESM.pdf]

Description of Additional Supplementary Files

File Name: Supplementary Movie 1
Description: A video of Dyneema polyethylene fibers that have been plasma dye coated with disperse red 1 acrylate. The halochromic response towards hydrochloric acid and ammonia gases is shown by successive exposure of the fibers to these gases.
